# Supplementary material for: Polyketide synthase gene clusters in the ericoid mycorrhizal fungus Oidiodendron maius: genome-wide prediction, expression profiling, and functional characterization of knockout mutants
Source: Front Plant Sci. 2026 Sep 16;17:1924430. doi: 10.3389/fpls.2026.1924430 (PMC13623903; doi:10.3389/fpls.2026.1924430)
Supplement: Supplementary file 1 [file SupplementaryFile1.zip › Supplementary Materials/Supplementary Folder 1.zip!/Oidma1_AssemblyScaffolds_Repeatmasked/knownclusterblast/cluster47/jgi.p_Oidma1_76913_mibig_hits.html]

| MIBiG Protein | Description | MIBiG Cluster | MiBiG Product | % ID | % Coverage | BLAST Score | E-value |
| --- | --- | --- | --- | --- | --- | --- | --- |
| AAC49195.1 | pathway\_specific\_transcription\_factor | BGC0000152 | Sterigmatocystin\_biosynthetic\_gene\_cluster | 34 | 105.925925926 | 172 | 1.1e-42 |
| CBF90101.1 | Putative\_Zn(II)2Cys6\_transcription\_factor\_(Eurofung) | BGC0001271 | Emericellin\_biosynthetic\_gene\_cluster | 34 | 111.604938272 | 163 | 4e-40 |
| AAS90051.1 | AflR | BGC0000009 | Aflatoxin\_biosynthetic\_gene\_cluster | 31 | 108.148148148 | 153 | 7.1e-37 |
| AAS90086.1 | AflR | BGC0000010 | Aflatoxin\_biosynthetic\_gene\_cluster | 32 | 107.901234568 | 152 | 9.3e-37 |
| AAS90026.1 | AflR | BGC0000008 | Aflatoxin\_biosynthetic\_gene\_cluster | 32 | 107.901234568 | 152 | 9.3e-37 |
| AAS90091.1 | AflR | BGC0000006 | Aflatoxin\_biosynthetic\_gene\_cluster | 32 | 107.901234568 | 152 | 9.3e-37 |
| AAS90003.1 | AflR | BGC0000007 | Aflatoxin\_biosynthetic\_gene\_cluster | 32 | 107.654320988 | 149 | 1e-35 |
| BAE71318.1 | regulatory\_protein | BGC0000004 | Aflatoxin\_biosynthetic\_gene\_cluster | 32 | 107.901234568 | 146 | 8.7e-35 |
| EAL89341.2 | C6\_transcription\_factor\_(AflR),\_putative | BGC0001403 | Trypacidin\_biosynthetic\_gene\_cluster | 33 | 108.148148148 | 144 | 1.9e-34 |
| AGO59037.1 | PtaR2 | BGC0000121 | Pestheic\_acid\_biosynthetic\_gene\_cluster | 31 | 105.432098765 | 125 | 1.6e-28 |
| ADM34145.1 | trancriptional\_activator | BGC0001084 | Notoamide\_/\_stephacidin\_biosynthetic\_gene\_cluster | 29 | 116.543209877 | 112 | 1.4e-24 |
| ACH72907.1 | AflR | BGC0000011 | Aflatoxin/sterigmatocystin\_biosynthetic\_gene\_cluster | 27 | 99.2592592593 | 107 | 3.4e-23 |
| ABK64185.1 | zinc\_finger\_transcription\_factor | BGC0001247\_c8 | Cercosporin\_biosynthetic\_gene\_cluster | 27 | 103.950617284 | 90 | 4.3e-18 |
